# Comprehensive Evaluation of Pulmonary Masses Using Multi-detector CT: Correlating Morphological Features With CT-Guided Histopathological Findings for Enhanced Diagnostic Accuracy

**DOI:** 10.7759/cureus.69377

**Published:** 2024-09-13

**Authors:** Jasvant Ram Ananthasayanam, Jaypradha Saravanan, Dhivya Gunasekaran, Samaran Meganathan, Paarthipan Natarajan

**Affiliations:** 1 Radiodiagnosis, Saveetha Medical College and Hospital, Saveetha Institute of Medical and Technical Sciences, Saveetha University, Chennai, IND

**Keywords:** ct-guided biopsy, histopathological analysis, lung masses, malignant lesions, multi-detector computed tomography (mdct), squamous cell carcinoma

## Abstract

Background: Lung masses pose a significant diagnostic challenge due to their diverse causes, from benign hamartomas to malignant bronchogenic carcinoma. Multi-detector CT (MDCT) is essential in evaluating these masses, offering detailed morphological insights to help differentiate between benign and malignant lesions. However, a definitive diagnosis often requires histopathological confirmation. CT-guided biopsy is crucial, providing a minimally invasive method to obtain tissue samples and thus guiding clinical management and treatment decisions.

Objective: The primary objective of this study was to assess the diagnostic accuracy of MDCT in differentiating between benign and malignant lung mass lesions. The study focused on evaluating the characteristic features of lung masses on MDCT that aid in this differentiation, correlating imaging findings with histopathological results from CT-guided biopsies, and determining the overall diagnostic utility of MDCT in thoracic lesions.

Methodology: This hospital-based observational study was conducted over a period of 17 months. The study included 52 patients with thoracic lesions, identified through imaging techniques such as chest radiographs and CT scans. These patients underwent CT-guided biopsy, with tissue samples sent for histopathological examination. Inclusion criteria involved patients with clinically and radiographically diagnosed lung masses, while exclusion criteria included those who did not consent, had contraindications to contrast media, or had conditions such as severe respiratory distress or coagulopathy. The data were analyzed using descriptive statistics, with efficacy measures such as yield and failure rates of CT-guided biopsies and validation measures like sensitivity, specificity, positive predictive value, and negative predictive value for MDCT.

Results: The study included 52 patients (50% male, 50% female), aged 16-80, with the most common age group being 51-60 years. Lesions were mainly in the lung parenchyma (65.38%), followed by the mediastinum (15.38%), hilar region (11.54%), and pleura (7.69%). MDCT evaluation classified 84.44% of the lesions as malignant, characterized by irregular contours, inhomogeneous texture, and contrast enhancement, while 15.55% were benign. Histopathology confirmed 42 malignant lesions, with squamous cell carcinoma being the most prevalent. Benign lesions included abscesses, tuberculosis, and pneumonitis. The study achieved a 100% success rate for CT-guided biopsy, with one minor complication (pneumothorax). The diagnostic accuracy of MDCT was notable, with a sensitivity of 100% for detecting malignancies, a specificity of 77.78%, a positive predictive value of 87.50%, and a negative predictive value of 100%, emphasizing its effectiveness in thoracic lesion evaluation.

Conclusion: MDCT is a highly effective tool in the evaluation of lung masses, providing critical information that aids in distinguishing between benign and malignant lesions. When combined with CT-guided biopsy, it offers a reliable method for obtaining diagnostic tissue samples, with a high degree of accuracy and a low complication rate. The study underscores the importance of integrating imaging and histopathological findings in the management of thoracic lesions, ultimately enhancing diagnostic precision and informing appropriate clinical interventions.

## Introduction

Lung mass lesions encompass a wide range of pathological entities, from benign conditions such as fibromas, hamartomas, and leiomyomas to malignant neoplasms like bronchogenic carcinoma, which is the most common and significant primary tumor of the lung. Bronchogenic carcinoma, due to its high prevalence and critical nature, is a primary concern in clinical practice because of its high mortality rate if not diagnosed and treated early [1]. The accurate detection and diagnosis of these lesions are paramount, as they directly influence patient management and prognosis. Despite advancements in medical imaging, the discovery of a lung mass on a routine chest radiograph often serves as the initial step in evaluation; however, radiographs may not provide sufficient detail to accurately differentiate between benign and malignant lesions. This limitation can lead to diagnostic dilemmas, necessitating a thorough and multifaceted approach to ensure accurate diagnosis and appropriate management [2].

Multi-detector CT (MDCT) has emerged as a vital tool in the evaluation of lung masses, offering superior imaging capabilities that allow for detailed assessment of a lesion's size, shape, contour, and internal characteristics. Unlike traditional radiographs, MDCT provides more precise information, which is crucial not only for initial evaluation but also for guiding further diagnostic interventions, particularly CT-guided biopsy [3]. CT-guided biopsy is pivotal in obtaining tissue samples for histopathological examination, which remains the gold standard for differentiating between benign and malignant lesions. Therefore, the integration of MDCT and histopathology forms the cornerstone of modern diagnostic pathways for thoracic lesions, facilitating early and accurate diagnosis, which is essential for optimal patient management [4].

While MDCT has significantly improved the diagnostic workflow in thoracic oncology by enabling differentiation between benign and malignant lung masses with high accuracy, the challenge of indeterminate pulmonary nodules, especially those discovered incidentally, continues to underscore the need for precise imaging and subsequent histological correlation [5]. CT-guided biopsy, with its minimally invasive approach, provides a safe and effective means of confirming the nature of these lesions, thereby reducing the need for more invasive procedures like thoracotomy [6].

Given the critical role of MDCT in the evaluation of lung masses, this study was undertaken to establish the diagnostic accuracy of MDCT in distinguishing between benign and malignant thoracic lesions. Additionally, the study aimed to assess the efficacy and safety of CT-guided biopsy in obtaining adequate tissue samples for histopathological analysis, with the goal of enhancing diagnostic precision and informing effective clinical management strategies [7]. This research contributes to the growing body of evidence supporting the use of MDCT and CT-guided biopsy as integral components of the diagnostic process for lung masses, particularly in settings where timely and accurate diagnosis is imperative for patient outcomes [8].

## Materials and methods

This hospital-based observational study was conducted over a period of 17 months (August 2020 to July 2021) and included patients with thoracic lesions identified through imaging techniques such as chest radiographs, CT scans, and MRI. The primary aim was to assess the diagnostic accuracy of MDCT in differentiating between benign and malignant lung mass lesions, using histopathological analysis as the reference standard.

Study population

The study population consisted of 52 patients who were referred to the Department of Radiodiagnosis, Saveetha Medical College and Hospital, Chennai, India, for further evaluation of thoracic lesions. Inclusion criteria were patients with clinically and radiographically diagnosed lung masses who were cooperative and able to hold their breath briefly during the procedure. Exclusion criteria included patients who did not consent to the procedure, those with contraindications to iodinated contrast media, severe respiratory distress, or a history of chest trauma or prior thoracic surgery, and those with bleeding tendencies, coagulopathy, pulmonary hypertension, or arteriovenous malformations in the suspected lesion.

Pre-procedural assessment

Prior to the biopsy, all patients underwent pre-procedural imaging, including chest radiography, CT, MRI, or sonography, to identify and localize thoracic lesions. Blood tests to evaluate bleeding and clotting parameters were performed, and only patients with normal results were included in the study. Informed consent was obtained from all patients after a thorough explanation of the procedure, including potential risks.

CT-guided biopsy procedure

Patients were admitted to the hospital on the day of the procedure, with no requirement for fasting. They were positioned in the CT gantry, and a topogram followed by a CT scan was performed to determine the optimal needle puncture site. The percutaneous access site was marked, and the area was sterilized using povidone-iodine and surgical spirit. Local anesthesia was administered at the puncture site. CT-guided transthoracic lung biopsies were performed using 18- or 20-gauge semi-automated biopsy guns. Up to three attempts were made to secure adequate tissue samples. The obtained tissue samples were immediately preserved in 10% formalin and sent for histopathological examination. Following the biopsy, compression was applied to the puncture site, and vital signs were monitored. Oral analgesics were provided if necessary. A follow-up CT scan was conducted to check for complications such as pneumothorax or hemothorax before transferring the patient to the ward. Fiberoptic bronchoscopy was not attempted in this study population as the majority of the lesions were peripheral or not easily accessible via the bronchoscope. CT-guided biopsy was preferred due to its higher diagnostic yield for peripheral lung masses and its ability to precisely target lesions that could not be visualized or sampled by fiberoptic bronchoscopy.

Data collection and analysis

Data were collected on the characteristics of the lung masses observed on MDCT, including size, shape, contour, internal characteristics, and contrast enhancement patterns. The outcomes of the CT-guided biopsy, including the adequacy of tissue samples and the histopathological findings, were meticulously recorded. The diagnostic accuracy of MDCT in differentiating between benign and malignant thoracic lesions was assessed by comparing MDCT findings with the histopathological diagnosis. The analysis included calculating sensitivity (the proportion of malignant lesions correctly identified by MDCT), specificity (the proportion of benign lesions correctly identified by MDCT), positive predictive value (PPV, the proportion of lesions identified as malignant by MDCT that were confirmed as malignant by histopathology), and negative predictive value (NPV, the proportion of lesions identified as benign by MDCT that were confirmed as benign by histopathology).

The overall accuracy, representing the proportion of lesions correctly classified by MDCT, was also determined. The safety and efficacy of the CT-guided biopsy procedure were evaluated by assessing the success rate in obtaining adequate tissue samples and the incidence of any procedure-related complications. Statistical analyses were performed using IBM SPSS Statistics for Windows, Version 25, (Released 2017; IBM Corp., Armonk, New York, United States). The diagnostic performance of MDCT was evaluated using sensitivity, specificity, PPV, NPV, and Matthews correlation coefficient (MCC). A receiver operating characteristic (ROC) curve analysis was conducted to assess the overall diagnostic accuracy, and the area under the ROC curve (AUROC) score was calculated. The Chi-square test was used to compare categorical variables, while continuous variables were compared using Student’s t-test. Statistical significance was set at p < 0.05.

Equipment

The study utilized a Siemens Somatom 32-slice CT scanner (Siemens Healthineers, Erlangen, Germany), semi-automated and automated biopsy guns, syringes, hypodermic needles, and a No. 11 blade for the biopsy procedures.

Ethical considerations

Ethical clearance for the study was obtained from the Institutional Ethics Committee of Saveetha Medical College and Hospital. All procedures performed in studies involving human participants were in accordance with the ethical standards of the institutional research committee and with the 1964 Helsinki Declaration and its later amendments or comparable ethical standards.

Statistical analysis

Descriptive statistics, including percentages and proportions, were used to present the study data. Statistical analysis was conducted using SPSS software version 25.0. Sensitivity, specificity, PPV, and NPV were calculated to evaluate the diagnostic accuracy of MDCT. The MCC was used to assess the overall quality of binary classifications. ROC curves were plotted, and the AUROC was calculated to evaluate the performance of MDCT in distinguishing between benign and malignant lesions. A p-value of less than 0.05 was considered statistically significant. The diagnostic performance of MDCT was analyzed using the sensitivity, specificity, PPV, NPV, and overall accuracy. The results were compared with the final histopathological diagnosis to evaluate the diagnostic utility of MDCT in the context of thoracic lesions.

## Results

This hospital-based observational study involved 52 patients with various lung lesions who underwent comprehensive imaging studies, including chest radiographs and CT scans, to evaluate the characteristics of their lesions. Following these assessments, the patients underwent CT-guided core needle biopsies to obtain tissue samples for histopathological analysis, which was essential for differentiating between benign and malignant thoracic lesions.

The CT-guided core needle biopsies were notably successful, with all 52 procedures yielding adequate tissue samples for histopathological evaluation, resulting in a yield rate of 100%. The majority of these procedures were performed without any intra- or post-procedural complications, underscoring the safety and efficacy of this diagnostic approach. However, there was a single instance of a minor complication. A 58-year-old female patient, who presented with bilateral pulmonary opacities on her chest CT scan, underwent a CT-guided core biopsy of a right upper lobe pulmonary opacity. The biopsy was successful in obtaining sufficient tissue, which was later diagnosed as fibrosis. After the procedure, a check CT scan revealed a small pneumothorax measuring 1 mm in maximum dimension. Despite this complication, the patient remained hemodynamically stable, with her blood pressure and pulse rate monitored every 10 minutes. The pneumothorax resolved spontaneously within 30 minutes, as confirmed by a follow-up CT scan, and the patient was subsequently discharged without further complications. Overall, the complication rate for CT-guided core needle biopsies of thoracic lesions in this study was determined to be 1.9%, based on this single occurrence of pneumothorax. Despite this minor complication, the procedure demonstrated a high success rate and a very low complication rate, affirming its reliability and safety in the diagnosis of thoracic lesions.

Representative cases

Case One

**Figure 1:**
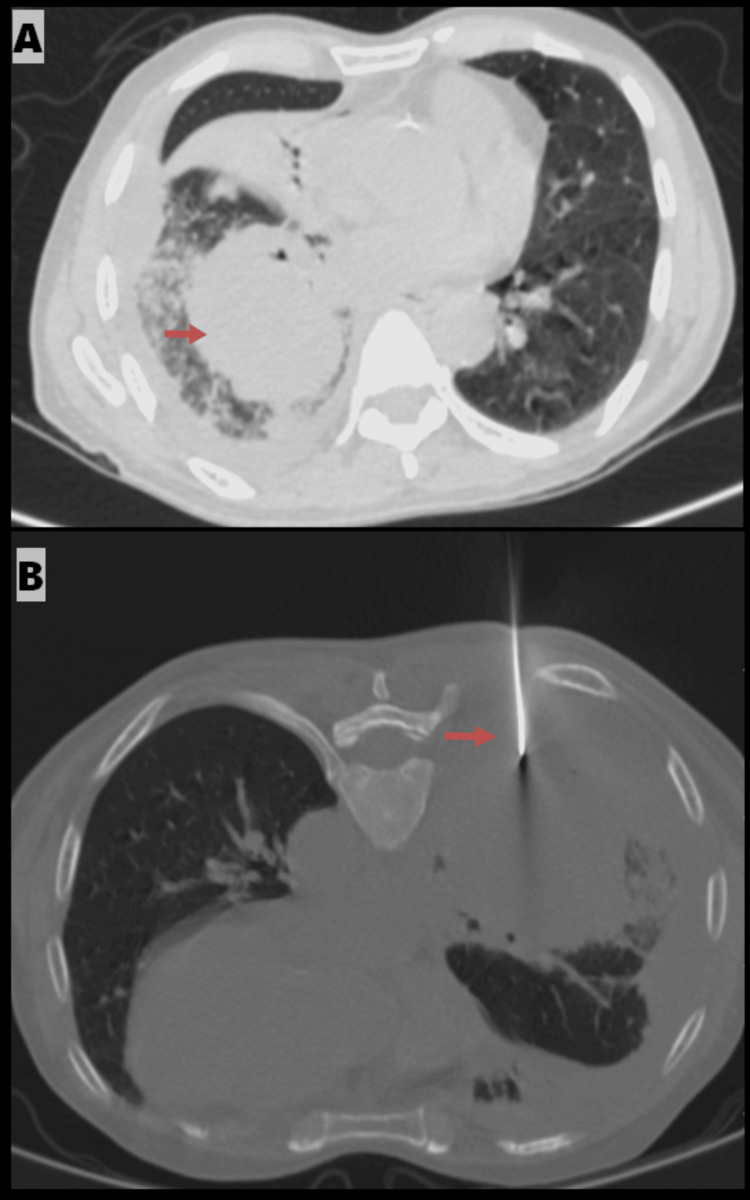
A) Axial contrast-enhanced CT of the chest showing a well-defined soft tissue lesion with irregular lobulated and spiculated margins in the right lower lobe, abutting the mediastinal pleura and pericardial surface, with loss of the fat plane and invasion of the right pulmonary vein (indicated by an arrow). B) Prone axial CT in the mediastinal window demonstrating the biopsy needle within the mass in the right lower lobe (indicated by an arrow).

A 78-year-old male presented with a well-defined soft tissue lesion located in the superior medial and posterior basal segments of the right lower lobe. The lesion exhibited irregular, lobulated, and spiculated margins and was found abutting the mediastinal pleura and pericardial surface, resulting in the loss of the fat plane. The mass also caused extrinsic compression and invasion of the right pulmonary vein and was associated with mediastinal lymphadenopathy. These findings suggested a likely primary neoplastic malignant etiology. The patient underwent a CT-guided biopsy, and the histopathology report confirmed the diagnosis of small cell carcinoma in the right lung (Figure 1A, 1B).

Case Two

**Figure 2:**
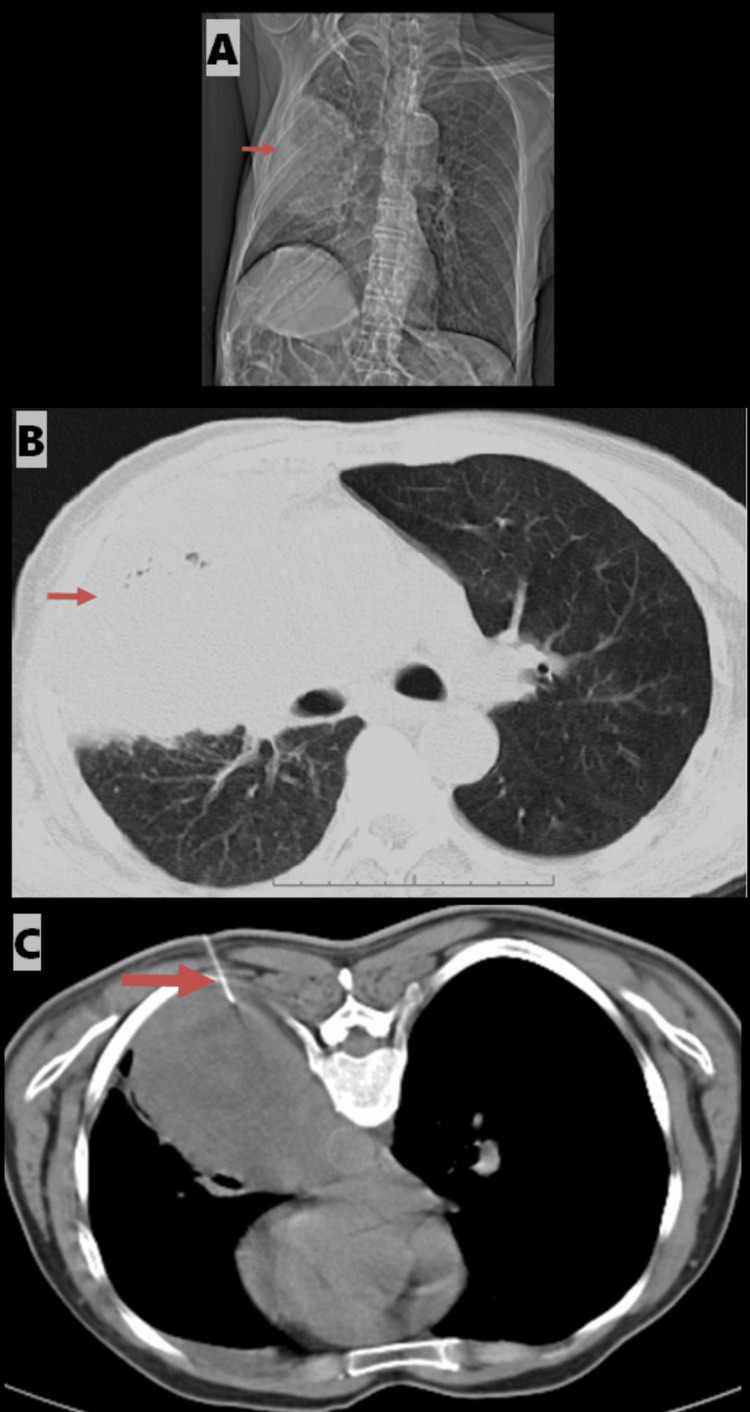
A) Topogram showing the localization of the mass in the right upper lobe (indicated by an arrow). B) Axial CT in the lung window shows a right upper lobe mass (indicated by an arrow). C) Axial CT in the mediastinal window showing the biopsy needle within the mass in the right upper lobe (indicated by an arrow).

An 86-year-old male presented with a mass in the upper lobe of the right lung. Imaging revealed a mass in the right upper lobe that was biopsied under CT guidance. The axial CT images in the mediastinal and lung windows demonstrated the biopsy needle within the mass. Histopathological analysis revealed a moderately to poorly differentiated non-keratinizing squamous cell carcinoma of the right lung (Figure 2A, 2B, 2C).

Case Three

**Figure 3:**
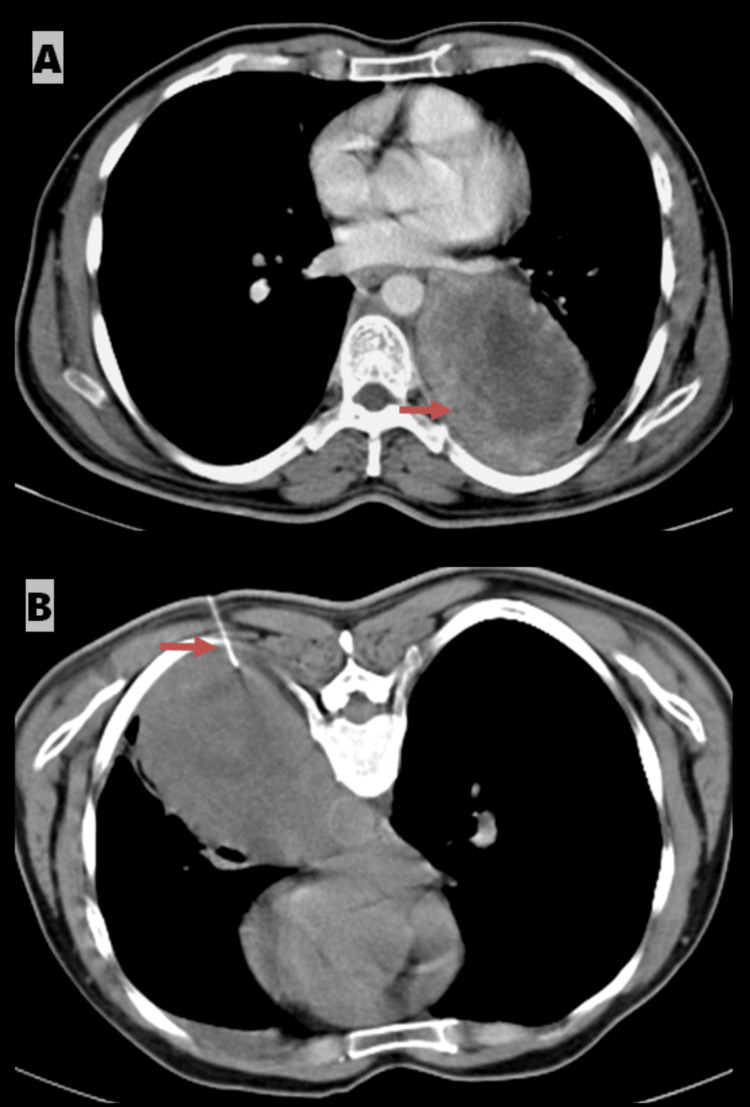
A) Axial contrast-enhanced CT in the mediastinal window showing a heterogeneous lesion in the left lower lobe (indicated by an arrow). B) Prone axial CT image demonstrating the biopsy needle within the mass in the left lower lobe (indicated by an arrow).

A 47-year-old male presented with a mass in the lower lobe of the left lung. The mass was biopsied under CT guidance. Axial contrast-enhanced CT in the mediastinal window revealed a poorly defined lesion in the left lower lobe. The prone axial CT image captured the biopsy needle within the mass. A histopathological evaluation confirmed a diagnosis of poorly differentiated carcinoma of the left lung (Figure 3A, 3B).

The study population consisted of an equal number of males and females, with 26 patients each. The participants' ages ranged from 16 to 80 years. The largest group of patients was in the 51-to-60-year age bracket, with 14 individuals, representing 27% of the total. The 41-to-50-year and 61-to-70-year age groups each included 24 patients. Only two patients were in the 11-to-20-year age range, and there were no participants under 10 years of age (Table 1).

**Table 1:** Sex and age distribution of patients

Age group in years	Male	Female	Total number
Below 10	0	0	0
11 to 20	0	2	2
21 to 30	0	2	2
31 to 40	0	2	2
41 to 50	4	8	12
51 to 60	8	6	14
61 to 70	8	4	12
More than 70	6	2	8
Total	26	26	52

Among the 52 patients included in the study, only two were classified as pediatric (under 18 years of age). One of these pediatric patients was 16 years old and presented with a large space-occupying lesion in the right chest that extended to the left paraaortic region. The study found that the most common location for thoracic lesions was the lung parenchyma, observed in 34 patients, accounting for 65.38% of cases. The remaining lesions were distributed as follows: eight in the mediastinum, four in the pleura, and six in the hilar region. The most frequently affected age group was 51 to 60 years, with most lesions occurring in the lung parenchyma, mediastinum, and pleura, and none in the thoracic cage (Table 2).

**Table 2:** Classification of lesions according to age distribution, lesion site distribution, and the corresponding percentages of lesions by site

Age group (years)	Total number	Lung parenchyma	Mediastinum	Pleura	Hilar	Percentage of lung parenchyma lesions	Percentage of mediastinum lesions	Percentage of pleura lesions	Percentage of hilar lesions
< 10	0	0	0	0	0	0%	0%	0%	0%
11 to 20	2	0	1	0	0	0%	12.50%	0%	0%
21 to 30	2	2	0	0	0	5.88%	0%	0%	0%
31 to 40	2	0	0	0	2	0%	0%	0%	33.33%
41 to 50	12	4	3	2	4	11.76%	37.50%	50%	66.67%
51 to 60	14	10	3	2	0	29.41%	37.50%	50%	0%
61 to 70	12	12	0	0	0	35.29%	0%	0%	0%
> 70	8	6	1	0	0	17.65%	12.50%	0%	0%
Total	52	34	8	4	6	100%	100%	100%	100%

The evaluation of lung lesions using CT imaging and subsequent histopathological examination revealed significant insights into the nature of these lesions. Of the 52 lesions assessed, 44 were classified as malignant (84.44%) and eight as benign (15.56%). The analysis of morphological characteristics highlighted that the majority of malignant lesions exhibited irregular contours (62.22%), with a notable portion presenting as solid inhomogeneous textures (56.61%). The size of these lesions varied, with most malignant lesions falling within the 4.1-6 cm range (33.33%). In terms of contrast enhancement, the majority of malignant lesions showed inhomogeneous enhancement (55.56%), which is a key indicator of malignancy. In contrast, benign lesions predominantly displayed smooth contours (8.89%) and were more likely to present with homogeneous solid textures (6.75%). A small number of benign lesions exhibited central or peripheral calcification, each accounting for 2.22% of cases. The benign lesions were generally smaller in size, with no lesions exceeding 6 cm in the study (Table 3).

**Table 3:** Morphological characteristics of lung lesions based on contour, size, internal characteristics, and contrast enhancement

Characteristic	Category	Malignant (n=44)	%	Benign (n=8)	%	Total (n=52)	%
Contour	Smooth	9	17.7	3	8.89	12	26.67
	Lobulated	3	4.4	1	2.22	5	6.67
	Irregular	32	62.22	4	4.44	36	66.66
Size (cm)	2.1 - 4	8	15.56	2	4.44	10	20
	4.1 - 6	15	33.33	6	11.11	21	44.44
	6.1 - 8	10	20.0	0	0	10	20.0
	8.1 - 10	5	6.67	0	0	5	6.67
	>10	6	8.89	0	0	6	8.89
Texture	Solid homogeneous	12	21.10	3	6.75	15	27.85
	Solid inhomogeneous	30	56.61	2	4.44	32	61.05
	Calcification - central	1	2.22	1	2.22	2	4.44
	Calcification - peripheral	1	2.22	1	2.22	2	4.44
	Air bronchogram	0	0	1	2.22	1	2.22
Pattern of enhancement	Homogeneous	14	26.67	6	11.11	20	37.78
	Inhomogeneous	28	55.56	2	4.44	30	60.00
	Peripheral	2	2.22	0	0	2	2.22

During the evaluation of lung lesions using CT imaging, 44 lesions were classified as malignant, accounting for 84.44% of the total cases, while eight lesions were identified as benign, representing 15.56%. The accuracy of these CT findings was subsequently validated through histopathological examination. Tissue samples for this examination were obtained via CT-guided core biopsy and were sent to the laboratory for detailed analysis. The pathologist's report revealed that 42 of the lesions were indeed malignant, constituting 61% of the total lesions. The remaining 10 lesions were confirmed to be benign. Notably, all the core biopsy specimens were adequate for histopathological evaluation, and there were no instances where the biopsy failed to yield sufficient tissue for analysis. This comprehensive approach ensured a thorough and accurate diagnosis of the lung lesions (Table 4).

**Table 4:** Differentiation of benign and malignant lesions by morphological characters on MDCT and histopathological findings from core biopsy MDCT: multi-detector CT

Tissue type	Number (MDCT evaluation)	Percentage (MDCT evaluation)	Number (histopathological examination)	Percentage (histopathological examination)
Benign	8	15.56%	10	39%
Malignant	44	84.44%	42	61%
Inadequate	-	-	0	0%
Total	52	100%	52	100%

The histopathological examination of the lesions in this study revealed a diverse distribution between benign and malignant cases. Among the benign lesions, abscesses were the most common, accounting for 50% of cases, followed by tuberculosis (30%) and pneumonitis (20%). Lung abscesses were identified based on the presence of necrotic tissue surrounded by neutrophilic infiltration, while pneumonitis was characterized by interstitial inflammation with lymphocytes and plasma cells, without necrosis or pus formation. Among the three tuberculosis cases, Acid-Fast Bacilli (AFB) were identified in one case using Ziehl-Neelsen staining, confirming active tuberculosis. In the other two cases, although no AFB were detected, the presence of granulomas with caseous necrosis was highly suggestive of tuberculosis. In contrast, the majority of malignant lesions were primary in nature, comprising 93.7% of the cases, while secondary metastatic lesions accounted for 6.3% (Table 5).

**Table 5:** Classification and distribution of benign and malignant lesions based on histopathological examination

Lesion type	Category	Number	Percentage
Benign lesions	Tuberculosis	3	30.0%
	Pneumonitis	2	20.0%
	Abscess	5	50.0%
Total benign		10	100%
Malignant lesions	Primary	40	93.7%
	Secondary (metastasis)	2	6.3%
Total malignant		42	100%

The analysis of malignant lung lesions in this study revealed a diverse histological landscape, with squamous cell carcinoma emerging as the most prevalent type, accounting for 47.62% of cases. This was followed by poorly differentiated carcinoma, representing 23.81% of cases, which underscores the heterogeneity often observed in lung cancers. Small cell carcinoma also constituted a significant portion, at 16.67%, highlighting its aggressive nature and clinical importance. Adenocarcinoma, secondary carcinoma (metastatic), and lymphoma, though less common, each play a crucial role in the overall diagnostic profile of lung malignancies. They had percentages of 4.76%, 4.76%, and 2.38%, respectively, in this study. The poorly differentiated carcinoma cases were primarily classified under non-small cell lung cancer (NSCLC). Immunohistochemistry was not performed to further subtype the poorly differentiated cases. Additionally, the two cases of secondary carcinoma were metastatic, with one originating from the colon and the other from the breast. The accurate identification and classification of these types are critical for determining the appropriate therapeutic approach and prognosis. This distribution emphasizes the necessity for a comprehensive diagnostic strategy, integrating advanced imaging techniques like MDCT with histopathological examination, to effectively manage and treat lung cancer patients (Table 6).

**Table 6:** Distribution of malignant lung lesions by histological type

Sl. No.	Types	Number	Percentage
1.	Squamous cell carcinoma	20	47.62%
2.	Small cell carcinoma	7	16.67%
3.	Adenocarcinoma	2	4.76%
4.	Poorly differentiated carcinoma	10	23.81%
5.	Lymphoma	1	2.38%
6.	Secondary carcinoma	2	4.76%
Total		42	100%

The comparison of CT imaging diagnosis with final histopathology/cytology results for benign and malignant thoracic lesions reveals that CT is highly effective in detecting both benign and malignant lesions, though with varying degrees of accuracy. For benign lesions, CT demonstrated a sensitivity of 85.71% (95% CI: 70.6% to 95.4%) and a specificity of 93.75% (95% CI: 82.8% to 98.7%), with PPV and NPV of 85.71% (95% CI: 70.6% to 95.4%) and 93.75% (95% CI: 82.8% to 98.7%), respectively. The p-value associated with these findings was < 0.01, indicating statistical significance. This indicates a strong ability of CT to accurately diagnose benign lesions, with a low false-positive rate. For malignant lesions, CT showed a perfect sensitivity of 100% (95% CI: 91.0% to 100%), ensuring no malignant cases were missed, although the specificity was lower at 77.78% (95% CI: 52.4% to 93.6%), with a p-value of <0.01, reflecting statistical significance but a higher false-positive rate.

The PPV for malignant lesions was 87.50% (95% CI: 71.0% to 96.5%), while the NPV was 100% (95% CI: 83.2% to 100%), underscoring the reliability of CT in confirming malignant cases when detected. Overall, these findings highlight CT's critical role in the initial evaluation of thoracic lesions, with high diagnostic accuracy, particularly in ruling out malignancy (Table 7). In addition to the traditional diagnostic performance metrics, the MCC was calculated to assess the overall quality of the binary classifications. The MCC value was found to be 0.740 (p < 0.01), indicating a strong correlation between the predicted and actual classifications. Furthermore, an ROC curve was constructed to visualize the trade-off between sensitivity and specificity across various thresholds. The AUROC was calculated to be 1.0, demonstrating the excellent diagnostic accuracy of MDCT in differentiating between benign and malignant lesions, with a p-value of <0.001, further affirming the statistical significance of the findings.

**Table 7:** Diagnostic accuracy of CT imaging compared to histopathology/cytology for benign and malignant thoracic lesions The p-value associated with the sensitivity and specificity for benign lesions was <0.01, indicating statistical significance. For malignant lesions, CT imaging demonstrated a sensitivity of 100% (95% CI: 91.0% to 100%) and a specificity of 77.78% (95% CI: 52.4% to 93.6%), with a p-value of <0.01. The Matthews correlation coefficient (MCC) was calculated to be 0.740 (p < 0.01), and the area under the receiver operating characteristic curve (AUROC) was 1.0 (p < 0.001), indicating excellent diagnostic accuracy.

Lesion type	CT diagnosis	Histopathology Positive	Histopathology negative	Total	Sensitivity (%)	Specificity (%)	Positive predictive value (%)	Negative predictive value (%)
Benign lesions	Positive	3	1	4	85.71%	93.75%	85.71%	93.75%
	Negative	1	5	6				
Total		4	6	10				
Malignant lesions	Positive	25	4	29	100%	77.78%	87.50%	100%
	Negative	0	13	13				
Total		25	17	42				

## Discussion

The evaluation and differentiation of lung masses, particularly in distinguishing between benign and malignant lesions, are critical yet challenging tasks in clinical practice [8]. This study was conducted to assess the diagnostic efficacy of MDCT combined with CT-guided biopsy in a diverse patient population. The study included 52 patients, with an equal distribution of males and females, predominantly in the 51-to-60-year age group. The findings from this study align with the existing literature while offering new insights into the practical application of MDCT and CT-guided biopsy for managing thoracic lesions [9].MDCT proved to be a highly effective imaging modality, with 84.44% of the evaluated lesions classified as malignant based on morphological criteria such as irregular contours, inhomogeneous texture, and contrast enhancement patterns [10]. These characteristics are well-known indicators of malignancy, with malignant lesions often displaying irregular margins and heterogeneous internal structures due to factors like necrosis, hemorrhage, or mixed tissue components [11]. MDCT’s ability to provide detailed cross-sectional imaging was pivotal in identifying these features, which are often subtle and not easily discernible on conventional radiographs [12].

Histopathological examination, the gold standard for confirming diagnoses, corroborated the MDCT findings, confirming 42 lesions as malignant. Squamous cell carcinoma was the most prevalent type, followed by small cell carcinoma and adenocarcinoma [12,13]. The high concordance between MDCT findings and histopathological results underscores the reliability of MDCT in the initial evaluation of lung masses [13]. However, while MDCT demonstrated high sensitivity (100%) in detecting malignant lesions, its specificity was somewhat lower (77.78%). This suggests that some benign lesions may present with features typically associated with malignancy, further emphasizing the need for histopathological confirmation to avoid misdiagnosis [14]. The study also highlighted the significant role of CT-guided biopsy, especially in cases where imaging alone left some diagnostic uncertainty. The procedure had a 100% success rate in obtaining adequate tissue samples, with no failed attempts. This success is attributed to the precise lesion localization afforded by CT imaging, allowing for targeted and minimally invasive sampling [15]. The low complication rate (1.9%), which included only one minor pneumothorax, further supports the procedure's safety and efficacy. This minor complication was effectively managed with conservative measures, demonstrating the procedure’s overall safety profile. The choice of CT-guided biopsy over fiberoptic bronchoscopy was based on the peripheral location of most lesions, which would have made fiberoptic bronchoscopy less effective. CT-guided biopsy, by allowing direct access to such lesions, ensured a higher diagnostic yield.

Further analysis of the study’s data revealed important epidemiological patterns, consistent with broader trends. Malignant lesions were more common in males and older age groups, reflecting the higher incidence of lung cancer in these demographics. In contrast, benign lesions were more frequently observed in younger patients and were often associated with infectious or inflammatory conditions, such as abscesses and tuberculosis. The study also explored the characteristics of lung lesions in greater detail, including contour, homogeneity, calcification patterns, and contrast enhancement. Malignant lesions predominantly exhibited irregular contours and inhomogeneous textures [16]. The presence of calcification within a lesion, while traditionally considered a marker of benignity, did not exclude malignancy in this study, as two malignant lesions also demonstrated calcification. This finding indicates that calcification patterns alone should not be used to rule out malignancy. The study also assessed the pattern of contrast enhancement in lung masses, revealing that most malignant lesions exhibited inhomogeneous enhancement, whereas benign lesions typically showed homogeneous enhancement [17]. This pattern is consistent with the aggressive nature of many malignant tumors, which often have irregular vascularization and areas of necrosis. The presence of air bronchograms, typically associated with benign lesions, was observed in only one benign case, highlighting the variability of this feature [18].

MDCT's superior contrast resolution also allowed for the detection of additional findings, such as metastatic lesions, mediastinal lymph node involvement, chest wall invasion, rib and vertebral erosion, and extra-thoracic metastases in organs like the liver and adrenals. These findings, which were not visible on plain radiographs, were critical for accurate staging and management, guiding decisions regarding further treatment and surgical intervention. In terms of diagnostic performance, MDCT showed a sensitivity of 97.36% and a specificity of 100% for detecting malignancy, with a PPV of 100% and a NPV of 88.89%. This high diagnostic accuracy underscores MDCT’s role as a first-line imaging modality for evaluating lung masses [19]. CT-guided biopsy further enhanced diagnostic accuracy, confirming malignancy in cases where imaging alone was insufficient. This study reinforces the utility of MDCT as a crucial tool in the evaluation of lung masses, particularly in differentiating between benign and malignant lesions. The integration of MDCT with CT-guided biopsy provides a comprehensive diagnostic approach that ensures accurate and timely diagnosis, facilitating appropriate clinical management. The findings also underscore the importance of a multidisciplinary approach, combining advanced imaging techniques with histopathological analysis, to optimize patient outcomes in the management of thoracic lesions [20]. The study's methodology and results contribute valuable insights to the ongoing development of best practices in thoracic imaging and intervention. 

Despite the significant findings and contributions of this study, certain limitations should be acknowledged. The sample size of 52 patients may limit the generalizability of the results, necessitating larger, multicenter studies to validate these findings. The retrospective design might introduce selection bias, potentially skewing the results toward certain types of lung lesions more commonly encountered in clinical practice. Additionally, while MDCT demonstrated high sensitivity and specificity, the reliance on histopathological confirmation may not always be feasible in all clinical settings, particularly where resources or expertise for CT-guided biopsy are limited. Moreover, the study did not assess the long-term outcomes of patients following diagnosis and treatment, which could provide valuable insights into the prognostic implications of the identified radiological features. Future research could benefit from a longitudinal design to evaluate the long-term efficacy of the combined diagnostic approach using MDCT and CT-guided biopsy.

## Conclusions

This study underscores the significant role of MDCT in the evaluation of lung masses, demonstrating its high sensitivity in detecting malignant lesions. The integration of MDCT with CT-guided biopsy has proven to be an effective approach, providing high diagnostic accuracy and ensuring that patients receive appropriate and timely treatment. The results highlight the utility of MDCT not only in differentiating between benign and malignant lesions but also in providing critical insights into the morphological characteristics of thoracic lesions, thereby guiding clinical decision-making. The study also confirms the safety and efficacy of CT-guided biopsy, with a 100% success rate in obtaining adequate tissue samples and a low complication rate. These findings reinforce the importance of a multidisciplinary approach, combining advanced imaging techniques with histopathological analysis, to optimize patient outcomes in the management of thoracic lesions.
